# Classic and Variants APLs, as Viewed from a Therapy Response

**DOI:** 10.3390/cancers12040967

**Published:** 2020-04-14

**Authors:** Marie-Claude Geoffroy, Hugues de Thé

**Affiliations:** 1 Institut National de la Santé et de la Recherche Médicale (INSERM) U944, Equipe Labellisée par la Ligue Nationale contre le Cancer, 75010 Paris, France; marie-claude.geoffroy@inserm.fr; 2 Centre National de la Recherche Scientifique Unité Mixte de Recherche 7212, Institut Universitaire d'Hématologie (IUH), 75010 Paris, France; 3 Institut de Recherche Saint-Louis, Université de Paris, 75010 Paris, France; 4 Assistance Publique-Hôpitaux de Paris, Service de Biochimie, Hôpital St-Louis, 75010 Paris, France; 5 Collège de France, PSL Research University, INSERM U1050, CNRS UMR 7241, 75005 Paris, France

**Keywords:** leukemia, retinoic acid signaling, ATRA, ATO, PML, targeted therapy

## Abstract

Most acute promyelocytic leukemia (APL) are caused by PML-RARA, a translocation-driven fusion oncoprotein discovered three decades ago. Over the years, several other types of rare X-RARA fusions have been described, while recently, oncogenic fusion proteins involving other retinoic acid receptors (RARB or RARG) have been associated to very rare cases of acute promyelocytic leukemia. PML-RARA driven pathogenesis and the molecular basis for therapy response have been the focus of many studies, which have now converged into an integrated physio-pathological model. The latter is well supported by clinical and molecular studies on patients, making APL one of the rare hematological disorder cured by targeted therapies. Here we review recent data on APL-like diseases not driven by the PML-RARA fusion and discuss these in view of current understanding of “classic” APL pathogenesis and therapy response.

## 1. Introduction

**Figure 1:**
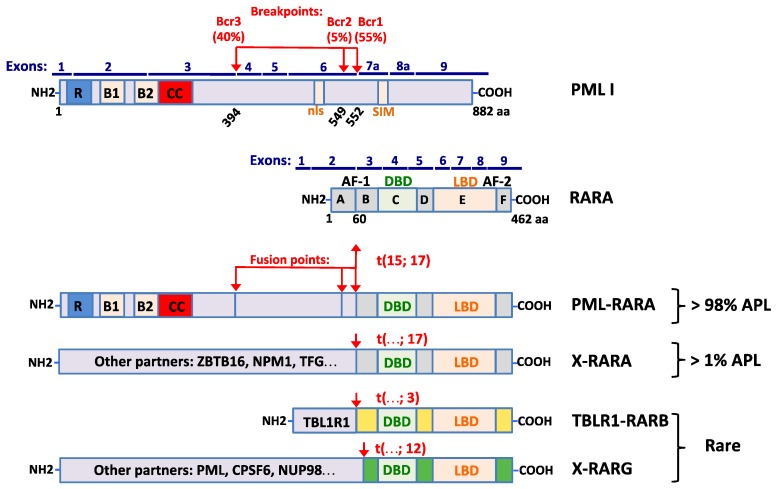
Structural organization of PML-RARA and other PML-independent RAR fusions in acute promyelocytic leukemia (APL) cells. Schematic representations of PML (isoform I), RARA and fusion partners of other retinoic acid receptors (RARB and RARG) before and after chromosomal rearrangements. Exons are shown in blue. The frequency of breakpoint cluster regions (Bcr) in APL is indicated in a percentage and fusion points are represented by a red arrow. The RING finger (R), B-boxes (B1 and B2), coiled-coil domain (CC), nuclear signal (nls) and sumo-interactif motif (SIM) in PML are represented by different colored boxes as well as functional domains in RARA: A-B: AF-1 transcriptional domain; C: DNA binding domain (DBD); D: hinge region; E: ligand binding domain (LBD), heterodimerization and AF-2 transactivation domain; F: unknown function.

Acute promyelocytic leukemia (APL) accounts for 10–15% of all acute myeloid leukemias (AML) with an annual incidence estimated at 0.0001% in Europe [1]. APL is a unique subtype of AML in which immature promyelocytes abnormally proliferate into the bone marrow. Classic APL is driven by a specific chromosomal translocation leading to the formation of PML-RARA, an oncogenic fusion protein found in over 98% of APL patients [2,3,4,5]. The resulting chimeric PML-RARA protein (Figure 1) promotes a clonal proliferation of myeloid precursors with a reduced differentiation capacity and acquisition of self-renewal. Pathogenesis of these “classic” APLs has been extensively reviewed elsewhere [6,7,8] and will only be sketched here. The World Health Organization (WHO) has revised the classification of AMLs, making APL with t(15; 17) (q24.1; q21.2), as a specific entity, but without taking in account the novel PML-independent RARs fusions [9]. Indeed, some other translocations driving the formation of fusion proteins have been described in very rare forms of leukemia with features of APL. While the genetics of these novel variant APL-like syndromes is now quite well delineated, their pathogenesis remains puzzling and treatment outcomes generally poor. Here, we review and put in perspective this recent literature on current understanding of genetic, pathogenesis and therapy response for these non “classic” APLs, stressing the role of global deregulated retinoic acid signaling in their pathogenesis.

## 2. Current Understanding of Classic APL Pathogenesis and Treatment Response

Pathogenesis of “classic” APLs was extensively reviewed elsewhere and will be only briefly summarized here. Fusion of PML to RARA yields an oncogenic transcription factor, which triggers both transcriptional deregulation of RARA and non-RARA target genes, together with disruption of PML nuclear bodies, the key regulators of senescence [10,11,12]. Multiple other features of PML-RARA were described, but their actual contributions to in vivo transformation remains unestablished. One of the most striking features of APL is its sensitivity to targeted therapies [6]. Indeed, APL has now become the most curable form of adult leukemia when treated by combined therapies with all trans-retinoic acid (ATRA) and arsenic trioxide (ATO). Clinical management of APL has been recently reviewed [13]. Major therapeutic advances were obtained in the ‘90s with the introduction of differentiation therapy with ATRA [14]. To avoid relapse that almost invariably occurs with ATRA monotherapy, ATRA plus anthracycline-based chemotherapy became the standard treatment for APL with complete remission rates to 90–95%, but delayed relapses occurred in many patients. First line administration of ATO in combination with ATRA radically changed the prognosis of a fatal disease into a fully curable one, even without chemotherapy [15,16,17,18]. The urgent necessity to make ATO available in all countries, in particular the oral form, which has proven its clinical utility in multiple trials, should be stressed in that respect [19,20].

ATRA acts at the transcriptional level to de-repress RARA target genes, yielding terminal differentiation of malignant promyelocytes into mature granulocytes [21]. Mechanistically, binding of ATRA to the RARA moiety of PML-RARA induces a conformational change that causes the dissociation of corepressors complexes and the recruitment of coactivators, activating gene re-expression. ATRA also acts on the stability of the PML-RARA protein by inducing its degradation in a proteasome-dependent manner, but also through proteolytic cleavage and autophagy-dependent mechanisms [22,23,24]. PML-RARA degradation per se results in the transcriptional reactivation of many differentiation genes, together with the reformation of PML nuclear bodies [25,26]. Arsenic binds directly onto specific cysteines residues located on zinc fingers, notably in the B2 domains of PML [27,28]. At therapeutic concentrations, ATO induces the sumoylation of both PML-RARA and PML, leading to their proteasomal degradation in an RNF4-dependant manner [29,30,31]. PML-RARA degradation by arsenic derepresses expression of differentiation genes, as well as allows reformation of PML nuclear bodies and restoration of senescence [32,33,34,35]. In addition, ATO targeting of the normal PML allele enforces NB nuclear bodies (NB) formation and contributes to therapy response. Indeed, mutations of the arsenic binding site of the unrearranged PML protein were discovered in therapy-resistant patients [36,37]. Thus, ATRA and ATO are targeted therapies directed onto the two constitutive moieties of the PML-RARA fusion. The dual targeting of PML-RARA (for destruction) and PML (for NB formation prior to catabolism) explains the potency of single agent arsenic for APL [6,38].

## 3. Mutations that Cooperate with Deregulated RARA Signaling to Drive APL and/or Therapy Resistance

Several recent studies have explored the gene mutations, which cooperate with PML-RARA to drive APL development or aggressiveness [39,40,41,42,43,44]. Many of these cooperating genes are also found in non-APL AMLs [42]. *FLT3* is the most frequent mutation in APL, present in roughly one third of patients. Other genes recurrently mutated included *WT1* (14%), *NRAS* (10%) and *KRAS* (4%). *MYC* amplification through trisomy 8 is also frequent (12% of cases). Advent of next generation sequencing (NGS) and whole exome sequencing of APL patients at diagnosis increased the variety of genetic alterations in APL, also demonstrating the existence of subclones [39,40,45]. Among new alterations, components of SWI/SNF complex, *ARID1A* (5%) and *ARID1B* (3%) genes were identified. Interestingly, genetic alterations commonly found in acute myeloid leukemia like *DNMT3*, *TET2*, *NPM1*, *IDH1* or *IDH2* are rarely detected, suggesting that PML-RARA exhibits a distinct transformation pathway among AML.

Mutations associated with relapse or therapy resistance have also been identified by these studies. Many mutations conferring resistance to ATRA or ATO are “on-target”, inhibiting direct binding of these agents onto PML-RARA, formally demonstrating that these agents are targeted therapies [46,47,48,49]. More recently, mutations on the arsenic-binding site of the normal *PML* allele have also been reported, demonstrating the key role of the normal *PML* gene in ATO response [36]. More broadly, independent studies have reported that activation of potent oncogenes at diagnosis was associated with ATRA plus chemotherapy resistance [40,50]. One particular case is *FMS-like tyrosine kinase 3* (*FLT3*), a receptor tyrosine kinase often mutated in AML. The most common mutations found in the internal tandem duplication (*FLT3-ITD*) region confers a high leukemic burden in APL and an adverse prognosis for patients treated by the ATRA/chemotherapy combination [51,52,53]. Recently, *FLT3-ITD* mutations were shown to severely blunt the ATRA response in animal models, precluding PML-RARA degradation and PML NB reformation [54], corroborating clinical studies. Yet, in mice models or patients, such resistance can be overcome by ATO, reinforcing the importance to use ATRA/ATO combination in high-risk APL patients with *FLT3* mutations [18,55,56].

## 4. Novel Retinoic Acid Receptors Fusions in APL

**Figure 2:**
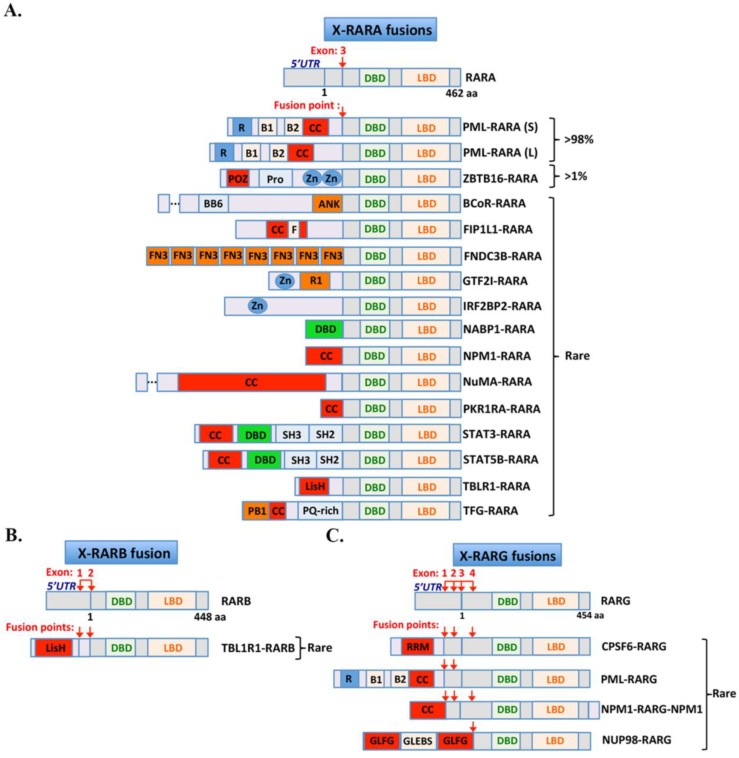
Schematic representation of the X-RARs fusions identified in APL: (**A–C**). Functional domains in X-RARA, X-RARB and X-RARG fusions proteins are represented by colored boxes. Exon and fusion points are indicated by a red arrow. Abbreviations: 5’-UTR: 5’ untranslated region; DBD: DNA binding domain; LBD: ligand binding domain; R: RING finger domain; B1 and 2: B box; CC: coiled-coil domain; POZ: BTB/POZ domain; Pro: proline-rich region; Zn: zinc finger domain; SH3: protein–protein interaction domain; SH2: docking domain for phosphorylated tyrosine residues; BB6: Bcl6- binding domain; ANK: ankyrin repeats; F: FIP1 binding domain for polymerase; FN3: fibronectin 3 domain; R1: putative HLH motif; LisH: lissencephaly type-1-like homology motif; PB1: Phox and Bem1 domain; PQ-rich: proline-glutamine-enriched domain; RRM: RNA recognition motif; GLFG: Gly-Leu-Phe-Gly repeats; GLEBS: Gle2/ Rae1-binding sequence.

Since the discovery of PML-RARA, more than a dozen diverse translocations involving RARA have been found in rare leukemia patients, often with typical morphological features of APL [57,58,59]. More recently, very rare fusions involving other retinoic acid receptors have also been described (Table 1, Figure 2) [60]. These results broaden the spectrum of APL-associated fusions and have important impact for our understanding of pathogenesis and treatment response.

### 4.1. PML-Independent RARA Fusions Involved in APL

In the classic t(15, 17)(q24; q21) with PML-RARA, the genomic breakpoint invariably occurred within the second intron of RARA. This led to in frame fusion with the PML gene (Figure 1). Similarly, in all others variants X-RARA translocations, partner genes X were fused to exon 3 of RARA. Interestingly, a feature common to most RARA partners resides in the presence of a dimerization domain and/or a DNA binding domain [91]. These may promote homo and heterodimerization of RARA and facilitate and relax specificity of X-RARA/RXRA complex binding onto DNA [92,93,94]. Therefore, abnormal X-RARA/RXRA tetramers may compete with normal transcription factor to promote oncogenic signaling. Indeed, dominant-negative RARA mutants alter lineage development of progenitors [95]. We shall review fusion proteins associated with retinoic acid receptors in APL-like syndromes, but will not discuss three ways of rearrangements implicating PML, RARA and a third gene.

Epidemiology points to a single rate limiting event, e.g., PML-RARA fusion [96]. Yet, existence of cooperative events has been well established in “classic” APLs (see above), demonstrating that a functional hierarchy exists, with PML-RARA playing a central role and many other alterations facilitating leukemia “progression”. Naturally, taken their rarity, cooperating events have not been well studied in variant APLs. In particular, any functional equivalent of the anti-senescence effects of PML disruption, if any, remain to be identified in those variants.

#### 4.1.1. ZBTB16-RARA t(11;17)(q23;q21)

The zinc finger and BTB domain containing 16 (BTB16), previously named PLZF, is a transcriptional factor involved in self-renewal and differentiation of stem cells [97]. This is the most frequent variant partner of X-RARA fusions, found in approximately 1% of APL patients. The N-term part of ZBTB16 (including the BTB/POZ domain and two Zn fingers) become fused to the DNA and ligand binding domains of RARA [61]. Reciprocal proteins RARA-ZBTB16 (comprising zinc fingers of ZBTB16 implicated in DNA-binding) are expressed and may contribute to leukemogenesis [98,99]. ZBTB16-RARA avidly recruits repressors such as SMRT, N-Cor and HDACs to repress RARA targets and block myeloid differentiation.

It was recognized early on that patients with ZBTB16-RARA fusions do not respond to ATRA or ATO [100]. Yet, at the molecular level, ATRA degrades the ZBTB16-RARA fusion, demonstrating that oncoprotein loss is not sufficient for differentiation or clinical response [33,34,101,102]. It was proposed that reciprocal RARA-ZBTB16 proteins confer some ATRA resistance by inducing upregulation of CRABPI, a retinoic acid binding protein involved in catabolism of retinoids [99]. Some clinical benefit was obtained when combining ATRA to other agents, including G-CSF or histone deacetylase inhibitors [103,104,105,106].

#### 4.1.2. BCoR-RARA t(X;17)(p11;q21)

The BCL6 corepressor (BCOR) is involved in multiple lymphomas [107,108]. BCOR is also a partner of PCGF1, a member of the noncanonical polycomb repressive complex 1 (PRC1.1). In BCOR-RARA fusion, the exon 3 of RARA is fused in frame with the first 12 exons of BCOR including the BCL6 binding domain. No reciprocal RARA-BCOR transcripts were detected. Two patients reached remission with an ATRA-chemotherapy regimen, including blast differentiation and one of them received ATO without any success at relapse [62,109].

#### 4.1.3. FIP1L1-RARA t(4;17)(q12;q21)

The FIP1-like 1 (FIP1L1) encodes a subunit of the CPSF (cleavage and polyadenylation specificity factor) complex. FIP1L1 may be implicated in leukemogenic fusion genes, notably in chronic eosinophilic leukemia where it may become fused with platelet-derived growth factor receptor alpha (PDGFRA) [110]. In the two patients where chimeric FIP1L1-RARA proteins were identified, the first 13 or 15 exons of FIP1L1 (including a homodimerization domain) were fused to RARA. FIP1L1-RARA functions as a transcriptional repressor in vitro and homodimerization of FIP1L1-RARA may be required [111]. In addition, the reciprocal RARA-FIP1L1 fusion gene was detected in both patients. The first patient achieved a complete remission by oral ATRA alone [63], while the second died of differentiation syndrome during induction therapy with ATRA [64].

#### 4.1.4. FNDC3B-RARA t(3;17)(q26;q21)

The FNDC3B (Fibronectin Type III Domain Containing 3B) is a regulator of adipocyte differentiation [112] and cell migration [113]. In APL, the first 24 exons of FNDC3B containing 8 fibronectin 3 (FN3) domains are fused in frame with RARA. FNDC3B-RARA fusion protein acts as a potent repressor on RARA targets genes. Two reciprocal RARA-FNDC3B transcripts were also detected. A unique patient with APL received ATRA and quickly developed the differentiation syndrome [65]. Addition of chemotherapy achieved complete morphological remission. Thus, ATRA can initiate in vivo differentiation of this APL-like syndrome.

#### 4.1.5. GTF2I-RARA t(7;17)(q11;q21)

The general transcription factor TFII-I (GTF2I) is an essential and pleiotropic transcriptional regulator whose defects are associated with neurodevelopmental defects and several types of cancers [114]. In APL, the translocation involving GTF2I and RARA creates a fusion between RARA exon 3 and the first 6 exons of GTF2I, including a leucine zipper (LZ) and putative helix-loop-helix (HLH). No reciprocal RARA-GTF2I transcript was detected. This unique patient received ATRA, which transiently improved coagulopathy but did not initiate morphological differentiation of leukemic blasts [66] followed by an ATRA-combined chemotherapy. The patient received then a combination of ATRA /ATO without any benefit. Upregulation of the RING finger protein 8 (RNF8) in GTF2I-RARA transfected cells was proposed to favor ATRA resistance [115].

#### 4.1.6. IRF2BP2-RARA t(1;17)(q42;q21)

Interferon regulatory factor 2 (IRF2) binding protein 2 (IRF2BP2) is a nuclear protein interacting with IRF2, a transcription factor involved in innate immunity [116]. IRF2BP2 zinc finger domain is fused with RARA exon 3 and reciprocal RARA-IRF2BP2 transcript could not be detected. IRF2BP2-RARA promotes immortalization of primary hematopoietic progenitors, which is abrogated with ATRA [67]. Among the five patients diagnosed with IRF2BP2-RARA APL-like syndrome, one was resistant to different treatments including ATRA alone [68]. Three patients showed a complete remission with ATRA-containing chemotherapy regimen [69,70,71]. Another one achieved differentiation and complete remission when ATRA was given as a single agent at diagnostic [67].

#### 4.1.7. NABP1-RARA t(2;17)(q32;q21)

The nucleic acid binding protein 1 (NABP1), previously named OBFC2A, encodes a single-stranded DNA binding protein domain involved in genomic stability [117]. In the context of the fusion, the first five exons of NABP1 including its DNA binding domain are fused to RARA exon 3. The unique patient reached complete remission with ATRA and chemotherapy, with some suggestions for differentiation induction [72].

#### 4.1.8. NPM-RARA t(5;17)(q35;q21)

Nucleophosmin (NPM1) is a nucleolar phosphoprotein involved in the pathogenesis of several hematological malignancies, including non-Hodgkin lymphomas where *NPM1* is a frequent translocation partner of the anaplastic lymphoma receptor tyrosine kinase (*ALK*) gene or AMLs where hotspot mutations of *NPM1* delocalize the protein to the cytoplasm and block differentiation [118,119]. In APL, the first four exons of *NPM1* including a hydrophobic oligomerization domain are fused to *RARA* exon 3 [73]. Reciprocal proteins RARA-NPM1 fusion proteins were also reported, but do not affect myeloid differentiation in cell culture [120]. NPM1 is a haplo-insufficient gene, so that loss of one allele may contribute to neoplastic transformation [121].

Among the dozen patients with NPM1-RARA, many are pediatric cases [73,122,123,124,125]. While they received induction with an ATRA-chemotherapy combination, most of them relapsed. Two patients received ATRA alone: one of them died of differentiation syndrome [123] and the other achieved complete remission prior to consolidation chemotherapy [126]. A rare case of atypical acute myelomonocytic leukemia was also reported [127], where ATRA combined to chemotherapy allowed durable remission. Thus, NPM1 fusions seem to exhibit significant ATRA-sensitivity.

#### 4.1.9. NuMA-RARA t(11;17)(q13;q21)

The nuclear mitotic apparatus protein 1 (NuMA) is an essential component for the formation and the maintenance of mitotic spindle poles during mitosis [128]. The first 20 exons of *NuMA* (including a long coiled-coil domain and a spindle binding domain) are fused to RARA [74]. No reciprocal RARA-NuMA proteins were detected. The unique patient achieved complete remission with ATRA [75].

#### 4.1.10. PRKAR1A-RARA t(17; 17)(q21; q24) or del(17)(q21q24)

The PRKAR1A gene encodes the regulatory subunit type Iα (RIα) of the cAMP-dependent protein kinase A (PKA). Its aberrant signaling leads to multiple skin abnormalities, diverse tumors and also infertility [129]. *PRKAR1* harbors a dimerization domain, which is fused to the DNA and ligand binding regions of RARA, as in others X-RARA translocations. Nevertheless, similar to STAT5B [130], dimerization domain of PRKAR1A is dispensable for transformation of murine myeloid progenitors [131]. Clinically, the unique patient shows a good clinical response to ATRA/chemotherapy [76].

#### 4.1.11. STAT5b-RARA t(17; 17)(q21; q21) or dup(17)(q12q21)

The signal transducer and activator of transcription (STAT5b) belong to a family of latent cytosolic transcription factors activated by cytokines or growth factors [132]. STAT5 is constitutively activated through multiple mechanisms during hematological transformation. In rare APL-like syndromes, the first 14, 15 or 16 exons of STAT5B (including coiled-coil, DNA binding and SH3/SH2 domains) are fused to RARA. No reciprocal RARA-STAT5b transcripts were found. At least 10 patients harboring STAT5B-RARA protein were reported with classic APL features [78,133,134,135,136,137,138]. Disease remained incentive to ATRA. No APL differentiation was observed with ATRA or ATO, while an ATRA and decitabine combination showed some response [139] so that this disease remains a therapeutic challenge [138].

#### 4.1.12. STAT3-RARA t(17;17)(q21;q21) or dup(17)(q12q21)

The signal transducer and activator of transcription 3 (STAT3) are phosphorylated in response to cytokines and growth factors [132]. Similar to STAT5 fusions, coiled-coil, DNA binding domains and SH3/SH2 domains of *STAT3* are fused to RARA. Clinically, the two patients reported were totally insensitive to ATRA and/or ATO treatment, even when combined to chemotherapy [77].

#### 4.1.13. TBLR1-RARA t(3;17)(q26;q21)

The transducin beta-like protein 1 (*TBL1*, also known as *TBL1X*) and its related gene (*TBLR1*, also known as *TBL1XR1*) are two exchange factors of NCoR/SMRT corepressors that regulate transcription [140]. For TBLR1-RARA chimeric proteins, the first five exons of *TBLR1* containing a LisH domain required for its dimerization were fused to *RARA* in two patients [79]. No reciprocal RARA-TBLR1 proteins were detected in patients. One of the two patients received ATRA-combined chemotherapy as induction treatment, which was interrupted because of toxicity. However, complete remission with an ATO and mitoxantrone combination was subsequently achieved [79]. A cryptic insertion of *RARA* (exon 3 to exon 9) was found between exon 5 and exon 6 of TBLR1 gene in a pediatric case [80]. This patient did not respond to an induction therapy with ATRA alone. TBLR1 was also identified as a translocation partner of RARB (see below).

#### 4.1.14. TFG-RARA t(3;14;17)(q12;q11;q21)

The tropomyosin-receptor kinase fused gene (*TRK*-fused gene, *TFG*) is a regulator of protein secretion [141]. *TFG* may be associated with lymphoma when fused with the *ALK* gene [142]. In APL, a complex chromosomal rearrangement creates a fusion between RARA exon 3 and the first 7 exons of *TFG* gene including a PB1 protein–protein-interaction domain and a coiled-coil motif. A unique patient with hypergranular morphotype showed a high sensitivity to ATRA alone reaching complete remission [81].

### 4.2. RARB and RARG Fusions Involved in APL-Like Leukemias

Two other members of the retinoic acid receptor superfamily (*RARB* and *RARG*) have now also been involved in leukemogenesis (Figure 1 and Figure 2, Table 1). All three receptors share a high sequence homology but have distinct transcriptional properties and expression patterns. Extensive studies in mice have pointed to some in vivo redundancy [143,144]. In hematopoiesis, RARA plays an important role on myeloid differentiation, while RARG is an important contributor of stem cell maintenance or inflammatory cytokine production [145]. When fused with PML all RARs members showed a similar oncogenic activity in in vitro assays [146]. However, as detailed below, unlike X-RARA fusion, most AML patients with RARG-rearrangement showed distinct clinical resistance to ATRA.

#### 4.2.1. TBLR1-RARB t(3;3)(q26;q24)

*TBLR1* gene, involved in rare *TBLR1-RARA* fusions, is also a translocation partner of RARB in APL. The same N-terminal part of *TBLR1* is fused to *RARB* exon 2 [82]. All patients were not only resistant to ATRA, but also to polychemotherapy.

#### 4.2.2. CPSF6-RARγ t(12;12)(q13;q15)

The cleavage and polyadenylation specific factor 6 (CPSF6) is a subunit of an RNA-binding protein complex. Different types of *CPSF6-RARG* fusion transcripts were found by whole genome sequencing or RNA-sequencing in five patients. All harbor the first four exons of *CPSF6* including a RNA recognition motif (RRM) fused in frame to either 5’UTR or exon 4 of *RARG* [83,84,85,147]. None of the patients did not show any ATRA sensitivity. The first one received ATRA and ATO followed by induction chemotherapy but died 37 days after diagnosis [84]. The two other cases report diagnosed with CPSF6-RARG showed also resistance to induction treatment with ATRA or ATRA plus arsenic [83,147]. Very recently, sensitivity of CPSF6-RARG to homoharringtonine and chemotherapy was reported in one patient [85].

#### 4.2.3. The NPM1-RARG-NPM1 (Karyotype Non-Determined)

The *NPM1* gene, already reported as a translocation partner of RARA, was also recently identified as a fusion partner of *RARG* [86]. Multiplex reverse transcription polymerase chain reaction (RT-PCR) and whole genome sequencing revealed insertion of *RARG* (5’UTR-exon9) within the *NPM1* gene to yield NPM1-RARG-NPM1 chimeric proteins. Partial deletion of *RARG* exon 10 causes a loss of 25 aa in the ligand binding domain of *RARG* that may impair ATRA binding, while the critical tryptophan residues implicated in *NPM1* nucleolar targeting are also altered. The unique patient was resistant to ATRA and ATO.

#### 4.2.4. NUP98-RARG t(11;12)(p15;q13)

NUP98 is a nucleoporin fused to a variety of partners in diverse myeloid and lymphoid malignancies [148]. All NUP98 fusions involve N-terminal GLFG repeats (for Gly-Leu-Phe-Gly) including a binding site for the mRNA export mediator *Gle2/ Rae1* (GLEBS domain) that provide a docking site for nucleocytoplasmic transport of RNA and protein. In the context of its fusion with *RARG*, the first 12 exons of *NUP98* are fused in frame with *RARG* exon 4, without reciprocal fusion products. Ex vivo transformation assays revealed that both GLFG repeats and DNA binding domain in *RARG* were required for the oncogenic activity of the chimeric protein [149]. ATRA or RXR agonists suppress transformation properties of NUP98-RARG in vitro and in vivo assays. Among the three patients reported to date, the first one had ATRA treatment for one day before chemotherapy initiation [87]. The two others patients [88,89] received ATRA/ATO without any success.

#### 4.2.5. PML-RARG t(12;15)(q13;q22)

A unique translocation t(12;15)(q13;q22) involving *PML* and *RARG* was reported in one case. Fusion breakpoints occurred within intron 3 of *PML* (like those observed in *PML-RARA* bcr3 fusions) and the 5’ untranslated region (UTR) of *RARG*. The unique patient with clinical features of classical APL received ATRA treatment for 9 days, but did not exhibit a differentiation [90]. Chemotherapy drove complete remission. ATO sensitivity was not documented. Overall, ATRA response was impossible to document in this setting.

### 4.3. Available Data for Therapy Response in Variant APLs

Constant involvement of RARA or other retinoic acid receptor super family members stresses the importance of deregulated retinoic acid signaling in driving APL pathogenesis. A key point is that therapy response is substantially different from that of classic APL, with inconsistent ATRA-sensitivity, but consistent ATO-resistance. Results from mouse studies have clearly outlined different types of responses to ATRA or ATO (differentiation and loss of self-renewal) corresponding to distinct molecular mechanisms. These often temporally overlap and synergize to enforce eradication of classic APL [6,33,34]. The first type of response is differentiation, driven by transcriptional reactivation of PML-RARA-silenced target genes. PML-RARA destruction (for example by ATO or shRNA) can similarly induce differentiation through promoter clearance [26]. However, differentiation is clearly not sufficient for the success of APL therapy [21]. Even AML dedifferentiation may occur and explain clinical relapses [150]. Apart from differentiation, a mechanistically distinct consequence of therapy is loss of APL self-renewal [33,34,35]. Clinically, this is reflected by a decrease in the burden of leukemic cells, restoration of normal hematopoiesis and eradication of the APL clone driving long-term survival. In classic APL, this is associated to restoration of PML nuclear bodies, well-known regulators of senescence, upon PML-RARA destruction [25,32].

Unlike PML-RARA, most of the novel retinoic acid receptors fusions associated with APL are clinically resistant to ATRA or ATO (Table 2). This is expected for ATO, which targets PML [32,151,152], but is more complex for ATRA. This reinforces the model that PML-driven senescence, initiated by PML-RARA degradation or arsenic targeting of PML, is an important contributor to a long-term clinical response [6,33]. Although clinical features and molecular characteristics of RAR partners are usually well documented for each case report, lack of information about blast decrease or differentiation upon ATRA or ATO treatments often precludes firm conclusions to be drawn, to mechanistically understand therapeutic failure. Moreover, it is not always easy to monitor APL differentiation and progressive clearance or to decipher the contribution of each mechanism to the therapy response. Naturally, as soon as targeted therapies are coupled with “classic” AML drugs (chemotherapy, demethylating agents and bone marrow transplantation) it becomes almost impossible to assess the contribution of ATRA or ATO, except if the clinical issue is rapidly fatal.

## 5. Are New Genetic Findings of APL-Like AMLs Shedding A New Light in Its Pathogenesis?

First, the variant APL-like associated translocation evidently points to the role of deregulated retinoid signaling at large in leukemia initiation. Significant evidence also points to enhanced repression and silencing of genes as a central and directly causative mechanism. Indeed, the most commonly affected retinoic acid receptor member, *RARA*, similar to the thyroid hormone receptors, is well known to bind more avidly corepressors than the two other members [153]. Then, ATRA dissociated the repressive SMRT/SIN/HDAC complex and allows recruitment of coactivators, so that the RARA ATRA couple behaves as molecular switch, with the ability to be repressive, neutral or activating. Second, variant fusions often contain identified repressive domains, very well characterized in the case of *ZBTB16* [154]. Similarly, the proposed central role of a dimerization domain provided by the fusion partner, remains to be experimentally substantiated for most APL-like syndromes [155], although it was suggested for some, such as *FIP1L1-RARA*. Ultimately, the nature and the respective importance of downstream target genes repressed by the RARA fusions remain largely unknown.

In trying to understand the physiopathology of variant translocations, one should not forget the literature linking myeloid differentiation to retinoic acid signaling and normal RARA. The historical model of ATRA-induced differentiation, HL60, exhibits *RARA* haplo-insufficiency, but no other genetic alteration [156]. Thus, fusions of *RARA* are not required for ATRA-induced differentiation and growth arrest of some AMLs. Conversely, ATRA-resistant HL60 mutants harbor mutations in RARA [157]. RARA regulates the normal differentiation of promyelocytes delaying it in RARA absence and accelerating it in the presence of ATRA [158]. Accordingly, dominant negative forms of RARA block myeloid differentiation of progenitors [95]. Actually, even overexpression of normal RARA induced blocks progenitors’ differentiation at the promyelocytic stage and promotes self-renewal [159]. Finally, recent studies in human AMLs have correlated the presence of RARA-associated super-enhancers to ATRA-induced differentiation [160]. This suggests that in the clinical setting, some AMLs exhibiting high RARA levels and critical RARA-associated transcriptional super-enhancer confers susceptibility to ATRA-induced differentiation. It is possible that this RARA super-enhancer is also linked to AML initiation. In that sense, addition of ATRA to chemotherapy in non-APL AMLs was proposed to have some clinical benefit [161,162]. Collectively, these observations highlight a complex and ill-understood relationship between retinoic acid signaling, normal myeloid differentiation, leukemic transformation and a potential benefit of ATRA signaling in AML cells, where RARA-mediated basal repression of retinoic acid signaling or its ATRA-triggered activation, seem to be a central theme, independently from fusion proteins.

While pathogenesis of classic APL clearly involves RARA- and PML-dependent features, it is possible that pathogenesis of some APL-like syndromes associated with rare X-RARA fusions is more closely related to immortalization by RARA overexpression [159], possibly not even requiring homodimerization through partner X. Indeed, basal RARA expression is low and the translocations not only fuse two genes, but also put the downstream gene under the control of the upstream gene promoter. In some cases, such as NPM1, these are very strong promoters [163], predicting supra-physiological levels of X-RARA expression, in contrast to PML-RARA. This may explain why some of these patients responded well to ATRA. Indeed, in either RARA-overexpressing cells or AML with RARA-associated super-enhancers, ATRA has a significant impact for survival or clonogenic activity [40,94,160]. Overall, we propose a working model wherein retinoic acid signaling has to reach a certain threshold to oppose transformation imposed by RARA overexpression or X-RARs fusion with fusion-acquired repressive properties. Note that some patients with classic APL were cured by retinoic acid alone, particularly with the liposomal form [164]. Since ATRA is expected to initiate degradation of any X-RARs fusion [23,101], it should probably be considered in the clinical management of any of the syndromes, because it should sharply down-regulate the steady state level of the oncogenic driver. Combinations, at least with classic chemotherapy, represents an attractive option, although demethylating agents or proapoptotic Bcl2 antagonists may also show some efficacy.

## 6. Conclusions: Deregulated Retinoic Acid Signaling in Other Malignancies?

Interestingly, recent studies have highlighted the existence of mutations of RARA, independently from its fusions, in other conditions [165], including rare subtypes of breast cancers [166]. Clinically, ATRA was proposed to enhance the efficacy of chemotherapy in AMLs, particularly in those bearing a mutation in NPM1 [161,162]. Several possible mechanisms where proposed, including NPM1c degradation [167,168] or interference with mutant IDH-mediated high ROS levels [169,170]. Finally, ATRA -and to a lesser extend ATO- were proposed to inhibit Pin-1, an enzyme implicated in the progression of multiple cancer types [169,171,172]. Clearly, any evidence for ATRA-reversible deregulation of retinoid signaling in other settings than APL could have considerable impact for patient care. Interestingly, some related findings were recently reported with vitD3 and its receptor [173]. Thus, links between nuclear receptor signaling and cancer is likely to be broader than the narrow example of APL and related APL-like diseases.

## Figures and Tables

**Table 1:** RAR partners causing APL and APL-like malignancies.

Fusion Protein	Incidence	Cytogenetic	References (1st Report When >10 Cases)
X-RARA			
PML-RARA	98%	t(15;17)(q22;q21)	[2]
ZBTB16-RARA	1%	t(11;17)(q23;q21)	[61]
BCoR-RARA	2 cases	t(X;17)(p11;q21)	[62]
FIP1L1-RARA	2 cases	t(4;17)(q12;q21)	[63,64]
FNDC3B -RARA	1 case	t(3;17)(q26;q21)	[65]
GTF2I-RARA	1 case	t(7;17)(q11;q21)	[66]
IRF2BP2-RARA	5 cases	t(1;17)(q42;q21)	[67,68,69,70,71]
NABP1-RARA	1 case	t(2;17)(q32;q21)	[72]
NPM1-RARA	>10 cases	t(5;17)(q35;q21)	[73]
NuMA-RARA	1 case	t(11;17)(q13;q21)	[74,75]
PRKAR1A-RARA	1 case	del(17)(q21q24)	[76]
STAT3-RARA	2 cases	t(17;17)(q21;q21)	[77]
STAT5b-RARA	>10 cases	t(17;17)(q21;q21)	[78]
TBLR1-RARA ^a^	3 cases	t(3;17)(q26;q21)	[79,80]
TFG-RARA	1 case	t(3;14;17)(q12;q11;q21)	[81]
X-RARB			
TBLR1-RARB *	3 cases	t(3;3)(q26;p24)	[82]
X-RARG			
CPSF6-RARG *	5 cases	t(12;12)(q13;q15)	[83,84,85]
NPM1-RARG-NPM1 *^,a^	1 case	ND	[86]
NUP98-RARG	3 cases	t(11;12)(p15;q13)	[87,88,89]
PML-RARG	1 case	t(12;15)(q13;q22)	[90]

ND: non-determined, * Fusion identified by whole genome sequencing, RNA-sequencing and RT-PCR, ^a^ cryptic insertion of RAR member within the partner gene (only in one case for TBLR1-RARA).

**Table 2:** ATRA/ATO therapy response for X-RARs fusions.

Fusion Protein	ATRA Response	ATO Response	Blast Decrease	Blast Differentiation	Self-Renewal of Bone Marrow
**X-RARA**					
PML-RARA	Sensitive	Sensitive	Yes	Yes	Yes
ZBTB16-RARA *	Resistant	Resistant	Yes	Yes	No
BCoR-RARA	Resistant	Resistant	ND	Yes ^a^	ND
FIP1L1-RARA	Sensitive ^a^	ND	Yes ^a^	Yes ^a^	Yes ^a^
FNDC3B-RARA	Uncertain	ND	No	Yes ^a^	No
GTF2I-RARA	Resistant	Resistant	No	No	No
IRF2BP2-RARA	Likely	Resistant	No	Yes ^a^	Yes ^a^
NABP1-RARA *	Uncertain	ND	No	Yes	ND
NPM1-RARA	Sensitive	ND	Yes	Yes	Yes
NuMA-RARA	Likely	ND	ND	Yes	ND
PRKAR1A-RARA	Uncertain	Uncertain	ND	Yes	ND
STAT3-RARA	Resistant	Resistant	No	No	ND
STAT5b-RARA	Resistant	Resistant	No	No	No
TBLR1-RARA	Resistant	Resistant	No	No	No
TFG-RARA	Sensitive	ND	Yes	ND	Yes
**X-RARB**					
TBLR1-RARB	Resistant	ND	No	No	No
**X-RARG**					
CPSF6-RARG	Resistant	Resistant	No	No	No
NPM1-RARG-NPM1	Resistant	Resistant	No	No	No
NUP98-RARG	Resistant	Resistant	No	No	No
PML-RARG	Resistant	ND	ND	No	ND

* ZBTB16-RARA (PLZF-RARA); NABP1-RARA (OBFC2A/RARA). ND: Non-determined; ^a^ (in 1 case). Uncertain: ATRA treatment combined with additional chemotherapy and/or stem cell transplantation.
